# Food insecurity in Venezuelan migrants in Trinidad and Tobago using the food insecurity experience scale

**DOI:** 10.3389/fpubh.2022.925813

**Published:** 2022-09-28

**Authors:** Arlette Saint Ville, Isabella Francis-Granderson, Brendon Bhagwandeen, Mizaaj Mohammed

**Affiliations:** ^1^Department of Geography, University of the West Indies, St. Augustine, Trinidad and Tobago; ^2^Department of Agricultural Economics and Extension, University of the West Indies, St. Augustine, Trinidad and Tobago

**Keywords:** food insecurity, Trinidad and Tobago, Venezuelan migrants, asylum seekers, FIES scale

## Abstract

Economic, political, humanitarian and health crises in Venezuela have resulted in mass out migration -thousands of Venezuelans emigrated to Trinidad and Tobago. However, little is known about their food security status in the host country. This study assessed the food security status among Venezuelan migrants and asylum seekers in Trinidad and Tobago and tested the validity of the online application of the food insecurity experience scale (FIES), a tool to measure food insecurity, in a small group. This convenience, cross-sectional study applied an online questionnaire to 433 Venezuelan migrants in Trinidad and Tobago in 2020. Snowball sampling was used to connect to migrants based on their access to locally-based NGO service providers, and organizations directly connected to the Venezuelan migrant community. Researchers applied the 12-month reference period to the FIES to measure food insecurity at the individual level. Descriptive analyses, Rasch modeling and binary logistic regression were conducted. Overall, 61.9% of respondents displayed behaviors characterized as severely food insecure. Significant differences in food security status were observed when categories of employment status (*p* = 0.032) and paying rent (*p* = 0.005) were considered. There were greater proportions of unemployed individuals who were severely food insecure (67.6%) compared to those who were employed (56.7%). There were greater proportions of individuals paying rent who were severely food insecure (62.6%) compared to those who were not paying rent (50.0%). Logistic regression with adjusted odds ratios and 95% confidence intervals revealed that food insecurity was less likely among migrants who were employed (OR 0.112, 95% CI 0.016–0.763) relative to those who were not employed, while food insecurity was more likely among migrants who were paying rent (OR 7.325, 95% CI 1.965–27.312) relative to those not paying rent. The FIES was consistent in assessing food security status. These findings provide a rapid assessment that can be used to galvanize international, national and community-level stakeholders to devise and target responses to assist migrants experiencing food insecurity.

## Introduction

Food security is multi-faceted and it is widely agreed that it exists when “all people, at all times, have physical and economic access to sufficient, safe and nutritious food that meets their dietary needs and food preferences for an active and healthy life” (1). Food insecurity is known to exist when there is limited or uncertain availability of nutritionally-adequate and safe foods, or limited or uncertain ability to acquire acceptable foods in socially acceptable ways (2).

While food insecurity can be measured at various levels, it impacts on various population subgroups, even within the same household differently. Research has shown that gender is a determinant of food security (3, 4). In particular, gender inequities experienced by women and girls have been shown to increase vulnerability to food insecurity (5). As a result of these inequities, across the world, women and girls represent ~60% of individuals experiencing chronic hunger (6). Understanding the over-representation of women and girls in the hungry and poor in various contexts and settings, especially in understudied small island developing states, is key to addressing socio-cultural and other discriminatory practices at various levels.

Although food insecurity exists among women and girls and the poor in developed and developing countries across the globe, it is also prevalent in marginalized subgroups such as migrants, refugees and asylum seekers (MRAS). This is as a result of the push factors that often led them to migrate and seek asylum in neighboring host countries, MRAS are often vulnerable from their source countries and remain vulnerable in their host countries. Studies in host countries identified factors linked to food insecurity among MRAS with economic constraints, limited knowledge about new foods, difficulty accessing grocery stores for food shopping, challenges with communicating in a new language, and the inability to comply with religious and cultural food dietary practices in a new setting or country (7–9). When facing new conditions in their host countries, migrants often continue and adopt new survival strategies to cope with food insecurity and so, develop new eating habits and skip meals (10). These strategies may further compromise their food security. Liden (11) found that refugees in Norwegian asylum centers avoid purchasing nutritious (more expensive) foods. These studies suggest that not only do these challenges increase the occurrence and severity of food insecurity among migrants, and result in their food needs often being unmet, which contributes to insufficient nutritional intake (11).

There are noted challenges to food security in Latin American and Caribbean countries that includes factors at the household and societal levels. Extant research on food security in developing nations evidence that food insecurity is linked to four factors namely violence (12–14), poverty (13), economic instability (13, 14) and climate change (13, 15). The interplay between environmental factors and food insecurity has been evident in predominantly El Salvador, Guatemala and Honduras (known as the Dry Corridor marked by lengthy dry spells and droughts). In their study, Barreto and Pisani (13) found that 47% of households in the Dry Corridor were moderately or severely food insecure because agriculture, the economy's backbone, was negatively impacted by irregular rainfall in these countries (13). Furthermore, 58% of households spent over two-thirds of their income on food before migrating, indicating high economic susceptibility to food insecurity (13). Such conditions in source countries of Latin America, that includes Venezuela, can contribute to food insecurity of households and drive migration into neighboring countries (14). Clearly, food insecurity in rural households that are dependent on rain-fed agriculture in the case of Latin America and the Caribbean is related to poverty, economic shocks and global environmental change.

Venezuela presents a unique case of political and economic decline in a non-conflict state in South America. While various authors describe differently the factors that gave rise to this decline, Bull and Rosales in their special issue, characterize it as involving “populism, democratic backsliding, the unraveling of a rentier economy, and how development can be put on reverse with informalization of the economy and dwindling economic growth” (16). These political factors gave rise to a 62% decline in gross domestic product (GDP) from 2013 to 2019 (16), and at the close of 2017, hyperinflation at a rate of more than 653% (17).

As conditions in Venezuela declined over time, the quantity and nature of the migrants changed. John (17), in a systematic review of Venezuela's economic and political crisis, describes migration flows (17). In the first wave, socialist-influenced policies drove out the wealthy, who left the country to safeguard their assets, while current waves involve those who are much poorer, but relatively well-educated. In the latter group, push factors involve survival as they emigrated to neighboring countries in search of jobs that often involve devaluing their skills to gain waged employment. These low-skilled jobs generate remittances needed to support family members left behind (17). Worsening economic conditions have resulted in irregular migration with humans trafficked for commercial sexual exploitation, and forced labor into neighboring countries.

While public health data has not been widely available since 2016, Page et al. noted that from 2012 to 2016, infant deaths in Venezuela increased by 63% and maternal mortality doubled (18). A recent survey noted that 80% of Venezuelan households were food insecure (19). Researchers suggest that there are crisis levels of acute malnutrition among children under five in vulnerable communities and growing malnutrition deaths.

Food insecurity can be considered as a push factor giving rise to large migrant flows to neighboring countries. From a population of some 24 million people, 15–20% of Venezuelans are believed to have fled the country and are currently displaced in the Latin American region (19). As of June 15th 2018, the United Nations High Commissioner for Refugees (UNHCR) registered 279,902 asylum applications by Venezuelans globally. Of these asylum seekers, 45% were located in neighboring Peru, 24% in the United States of America, 12% in Brazil and 6% in Panama (20). Due to the ongoing crises in Venezuela, millions of Venezuelans have fled their homes mainly to other regional Spanish-speaking countries that share land borders with Venezuela, and have even entered countries with maritime borders such as Trinidad and Tobago.

While the migration of Venezuelans to Trinidad and Tobago (T&T) is not a new phenomenon, the rise in numbers within a short time span in recent years, from fewer than 100 refugees in 2016 to over 6,480 Venezuelan migrants registered by the UNHCR in 2018 (21), has attracted both local and international attention. Currently, unofficial estimates suggest that Trinidad and Tobago hosts over 40,000 Venezuelans migrants (22). Anecdotal and official reports suggest that these numbers are expected to increase as Venezuelans are pushed out of the country because of threats to their lives, political freedom and safety, and limited access to basic human rights including health care, medicines, education and food (23).

Support for refugees and migrants in Trinidad and Tobago is provided by a number of organizations and implementing partners. The UNHCR operates a cash-based intervention system that helps migrants with money for food. Other organizations such as the International Organization of Migration (IOM) and Trinidad and Tobago Venezuelan Solidarity Network (TTVSOLNET) also organize distribution drives to provide migrants with food hampers. In 2017, the UNHCR and the Refugee Unit of the Immigration Division developed standard operating procedures to promote the provision of humanitarian aid, access to legal assistance, learning and other support services through their partner, Living Water Community (LWC), a non-governmental organization (NGO) (21). Other incentives geared toward empowerment and solidarity were conducted with academic, non-governmental and other groups, such as “Sticks in De Yard.” In addition, a number of other NGOs such as the Family Planning Association of Trinidad and Tobago (FPATT), Rape Crisis Society (RSC) and Families in Action (FIA), among others, were involved in protection responses on referral pathways inclusive of legal assistance, mental health, psychosocial support and sexual and reproductive health services (24).

Despite these critical services, Venezuelan migrants still face countless legal, social and economic challenges in Trinidad and Tobago. Despite the country acceding to the 1951 Geneva Convention on the Status of Refugees and its 1967 Protocol in November 2000 (25), the National Policy to Address Refugee and Asylum Matters in the Republic of Trinidad and Tobago (Refugee Policy), which was approved by its government in June 2014, has not been implemented. This 2014 Refugee Policy sets out a process that would transfer responsibility for Refugee Status Determination (RSD) procedures to the Trinidad and Tobago government. Until this takeover, the government has agreed to allow the UNHCR to conduct RSD for asylum seekers. However, only limited rights are received by those who are granted refugee status, with the right to access legal employment being excluded (21).

In the absence of asylum legislation, migrants who have fled persecution and unlawfully entered the country, or who have overstayed their lawful entry, are subject to repatriation, detention, or deportation, as well as hefty fines (21). The life and death imperative of fleeing means that migrants will subject themselves to any living and working conditions in order to remain outside of their country of persecution. Registration with LWC and the UNHCR does not prevent removal from Trinidad and Tobago (21). Consequently, many migrants seeking asylum live hidden lives, precariously housed and employed, without adequate food and nutrition, and in fear of detection by state authorities. These conditions can be seen as suboptimal and result in migrants living in fear without access to basic human rights, and has been described as being on the “frayed edge of the margins” (26).

In an attempt to alleviate the growing humanitarian concerns, in June 2019, the Government of Trinidad and Tobago implemented visa requirements for Venezuelans *via* the “Migrant Registration Framework,” a work permit exemption policy under the Ministry of National Security (27). Venezuelans who went through the 2-week registration exercise benefited from free emergency medical services at public health institutions including treatment for acute medical conditions such as accidents, injuries, asthma, heart attacks, strokes, diabetic comas, infectious disease and initial stabilization of fractures, public health promotions and immunization. Importantly, the registration exercise granted ~16,523 Venezuelan nationals stay-permits which allowed them to work for 6 months to 1 year (28), thereby enabling migrants to seek employment in the hospitality, entertainment, restaurant and construction industries.

In spite of registration and the granting of stay permits, obstacles persist as Venezuelan migrants seek to integrate in the Trinidad and Tobago society. Even though migrants employed in the country should be protected by local laws, and employees should pay them nothing less than the national minimum wage, this may not be the case in the informal sector. Other factors giving rise to delays with integration include language barriers, the lack of recognition of professional certificates and diplomas from Venezuela, limited access to specialized health care and administrative barriers which limit Venezuelan children and youth's access to school placement within the local public education system. Migrants also face exploitation of their labor, sexual exploitation of women and girls, stigma and xenophobia. These challenging situations may have an impact on food security and require in-depth study especially on the daily living conditions faced by migrant women and girls (21).

Importantly, the COVID-19 pandemic restrictions worsened conditions and placed migrants at increased risk. The closure of bars, restaurants and other establishments where many Venezuelan migrants usually found low-skilled employment in the informal sector led to loss of income and reduced ability to meet basic needs without any option of returning home, thus causing anxiety and despair for Venezuelans residing in Trinidad and Tobago. This situation also makes it more likely for migrants to become easy targets of criminal groups (24).

Despite fleeing their home country to escape food insecurity and poor living conditions, Venezuelan migrants nonetheless experience harsh conditions in the host country of Trinidad and Tobago that give rise to poor livelihood opportunities and lack of special temporary migrant status that relegates them to exploitative work conditions in the informal sector (21). This means that many of them are working without legal status and although employed (full-time or part-time) often in the informal economy, they experience unstable and insecure working conditions and low wages. In light of these expected developing country challenges, various NGOs provide food and assistance resources and work permits from the government (27). However, these Spanish-speaking MRAS still face numerous additional challenges that limit economic and social inclusion in the English-speaking country. Lacking meaningful opportunities for legal status, migrants are at risk of repatriation, deportation and detection by state authorities, are precariously housed, are without adequate food and nutrition and face limited education and employment opportunities. These factors, compounded by the impact of the COVID-19 pandemic, give rise to instability and uncertainty for Venezuelan migrants within the host country.

Prior studies have investigated the link between food insecurity and migration in Latin America and the Caribbean (13). However, little research has been conducted to identify Venezuelan asylum seekers' food security status in Trinidad and Tobago. This paper explores the food security status of Venezuelan migrants (a relatively small, marginalized group) in Trinidad and Tobago, delves into the associations of socio-demographic characteristics and food insecurity and tests the validity of a recent tool, the FIES, as an indicator to measure food insecurity in small groups.

## Materials and methods

### Study area, participants and data collection

**Figure 1:**
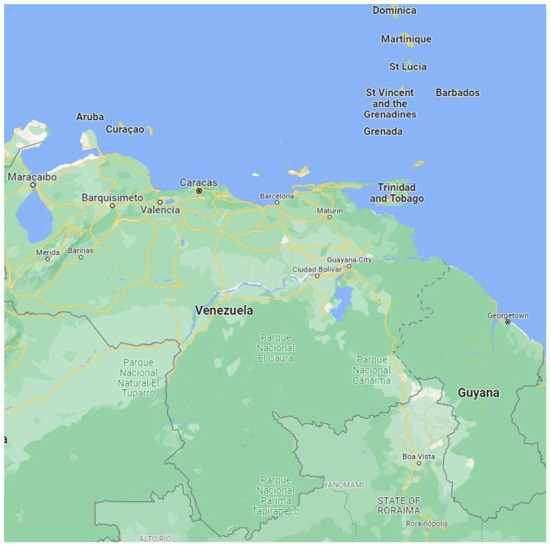
Map showing location of Trinidad and Tobago and Venezuela.

The research setting for this study is the twin-island English speaking country of Trinidad and Tobago. It is the most southernly island state in the Caribbean archipelago with a population of over 1,399,000 million (in 2020) with economic activity focused on oil and gas industries (29). Trinidad and Tobago lies north-east of the Spanish-speaking South American country of Venezuela, as shown in Figure 1 (30) and is located ~7 miles from Venezuela at its nearest point (31). The close geographical proximity of the two countries facilitates the migration of individuals from Venezuela to Trinidad and Tobago.

Although characterized as a high-income country with a GDP of 21.6 billion USD (32), Trinidad and Tobago has been designated as a small island developing state. Small Island Developing States (SIDS) are islands that experience unique vulnerabilities and are located in the Atlantic Ocean, Pacific Ocean, Caribbean Sea as well as the Indian Ocean (33). In the Caribbean region, SIDS such as Trinidad and Tobago are grappling with complex social and ecological challenges related to household food and nutrition insecurity, including high non-communicable disease rates, and rapid environmental change (34, 35).

A recent report assessed that 30% of Trinidad and Tobago's population reside in rural areas (36) and they are disproportionately affected by food insecurity. Further, a World Food Programme survey conducted in 2020 post-COVID estimated that a limited percentage of the population (6%) were involved in agriculture for commercial purposes (37). Overall, agricultural production has been in decline with agricultural land use reducing over time from 760 sq km in 1996 to 540 sq km in 2013 (36).

Trinidad and Tobago is susceptible to climate change as temperature and sea-level rise impacts soil quality and increases the occurrence of pest and disease outbreaks (36). As a result, agricultural production is currently and will probably continue to be disrupted by climate change. Climate events have a tremendous impact on agricultural production, since a third of all agricultural land is vulnerable to flooding. Overall, livelihoods in rural communities, experience economic and psycho-social decline from increasing climate events, and loss of crops that results in high uncertainty and unpredictable outcomes with negative impacts on rural food security (38).

Due to the COVID-19 outbreak and national lockdown in March 2020, data for this cross-sectional study were collected over a 4-week period from April 18^th^ 2020 to May 15^th^ 2020 using an online survey disseminated to participants *via* email and a mobile-based platform (WhatsApp). The Food Insecurity Experience Scale (FIES) (39) was used as an indicator. FIES was developed by the Food and Agriculture Organization of the United Nations (FAO) and launched globally in 2013. It was translated from English to Spanish for online deployment.

The study employed two sampling strategies. A convenience sample was generated based on migrants accessing local non-governmental organization (NGO) service providers. Firstly, a link was initiated by the Programme Manager of the Pan American Development Foundation (PADF) Trinidad and Tobago Office and shared with a total of 13 NGOs and service providers located across Trinidad and Tobago. After sharing with clients of various organizations, snowball sampling was employed to recruit additional migrants. Clients were requested to share the link to the study with their contacts within the migrant community. In total, responses came from a sample of *n* = 436 Venezuelan migrants resident in Trinidad and Tobago. Three persons were omitted from the subsequent analysis since they only provided a response for one sociodemographic question. This brought our final sample to *n* = 433 migrants.

Participant inclusion criteria entailed: (a) Venezuelan migrants aged 18 years and older; (b) currently resident in Trinidad and Tobago, (c) users of services (clients) from the PADF Trinidad and Tobago Office's partners and other NGOs and (d) social contacts of Venezuelan migrants who were users of services from one of the NGOs. Online distribution of the survey enabled the widespread recruitment of Venezuelan migrants across all four main areas (North, South, East and West) of the island of Trinidad. There were no surveys completed from the neighboring island of Tobago. Informed consent was obtained from all individual participants included in the study. This study was reviewed and approved by the Ethics Committee of the University of the West Indies, St. Augustine, Trinidad and Tobago.

### Data collection tool: The food insecurity experience scale

The FIES consists of eight questions arranged in order of increasing severity of food insecurity. It captures the latent trait of food insecurity (i.e., the condition of not having adequate access to food necessary to lead a normal and healthy life due to scarcity of money or other resources) in individuals, over the reference period of the past 12 months. The latent trait is assessed when participants self-capture their experiences, behaviors and perceptions (such as uncertainty) that are associated with having insufficient access to food as a result of a lack of resources (40, 41).

**Figure 2:**
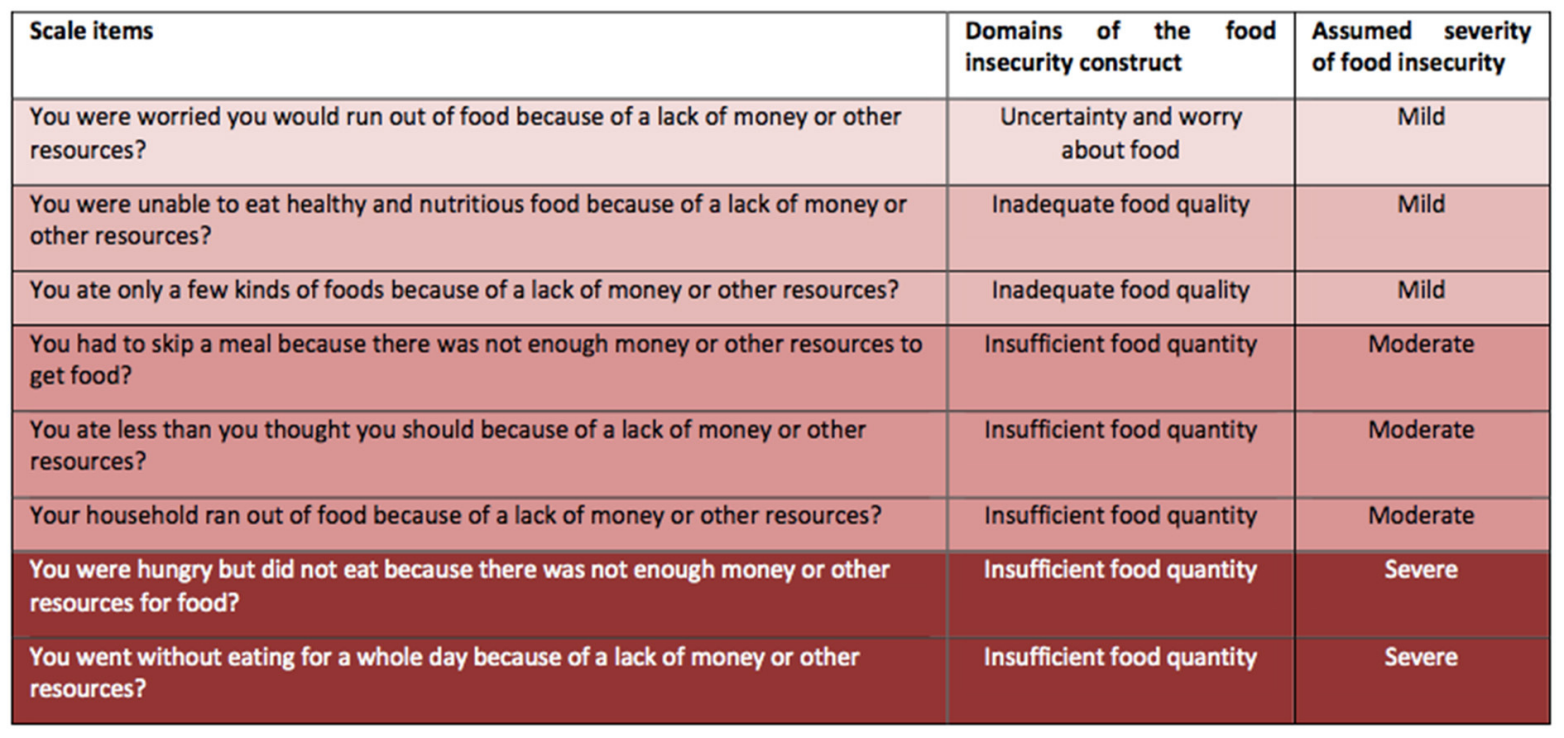
Food insecurity experience scale.

The FIES was used to categorize respondents along a food security continuum from “food secure” to “severely food insecure.” Respondents who answered “yes” to none of the questions were classed as “food secure.” Participants who answered “yes” to any of the questions were ranked into three categories of food insecurity (mild, moderate and severe food insecurity). These three categories of food insecurity correspond with food insecurity domains on the scale of uncertainty or worry about food running out, inadequate food quality and insufficient food quantity, respectively (24), as shown in Figure 2. The online survey incorporated socio-demographic and economic determinants of food insecurity such as gender, location of residence, employment status, number of work days per week, monthly income, payment of rent and presence of chronic illness.

### Statistical analysis

#### Descriptive analyses and associations

Descriptive analyses and binary logistic regression were conducted using IBM SPSS Statistics for Windows, Version 22.0 (42). Responses to the FIES questions were coded into binary categories, with affirmative responses given a value of one (1) and negative responses coded as zero (0). The sum of affirmative responses to the questions on the FIES was used to compute a raw score, which corresponded to a category of food insecurity: food secure to mild food insecurity (raw score 0–3), moderate food insecurity (raw score 4–6) and severe food insecurity (raw score 7–8). Unadjusted and adjusted odds ratios were presented. Associations between food insecurity and sociodemographic variables were observed using chi-square tests. For binary logistic regression, a binary measure of the severity of food insecurity (referred to as “food insecurity”) was created and coded as one (“1”) if an individual experienced moderate or severe food insecurity within the past 12 months and zero (“0”) if otherwise.

#### Rasch model: Internal validity test

Rasch model analysis was conducted on the eight (8) items in the FIES to detect differences in food security experiences across gender using R (43) and Microsoft Excel (44). The Rasch model is a type of Item Response Theory (IRT) measurement model that evaluates the quality of data generated by experience-based food insecurity scales and establishes the psychometric properties of the items in the FIES (45).

The Rasch model is based on three assumptions, namely: a) items discriminate equally, b) items are conditionally independent for respondents experiencing the same level of food insecurity and c) items measure the same construct (latent trait) of food insecurity. Equal discrimination was evaluated by an INFIT chi-square type statistic. INFIT means inlier-pattern-sensitive-type statistic and it is calculated as a mean of the squared residuals. Ideally, each item should have a value of one (1), but a range of 0.7–1.3 is deemed broadly acceptable (40). Assessment of conditional independence involved computing the relative item severity parameter from the correlation matrix of items. This assessment identified gaps in the items' structure in the scale by gauging the difficulty of each question relative to each respondent's food insecurity level (45). Accordingly, a food secure respondent is expected to answer affirmatively to less questions than a respondent experiencing mild, then moderate and ultimately severe food insecurity (41). Severity values reflect the position of the raw scores by using the natural log of the odds of probability within the food security questionnaire on a logit scale. A low severity value implies the category is in the mild domain, while a high severity value indicates that the category tends toward the severe domain (46). Lastly, a Wald test was applied to assess differences in the severities across gender.

## Results

### Characteristics of the study population

Table 1 describes the sociodemographic and economic characteristics of respondents. The majority of participants were female (67%, *n* = 290). They largely resided in the more urbanized areas of Trinidad, namely the Tunapuna-Piarco Municipality (19%, *n* = 84), the Borough of Chaguanas (17%, *n* = 72) and Diego Martin (12%, *n* = 51). The majority of respondents were employed (55.4%); were paying rent (95.6%) and belonged to households consisting of 3–5 members (57.3%). Only a limited number of participants had chronic illnesses (13.9%). More than one-fifth of the respondents earned a monthly income of 500–1,000 TTD or 75–150 USD (22%), while more than two-fifths worked 3–5 days each week (41%).

**Table 1:** Sociodemographic and economic characteristics of respondents.

**Variable**		***n***	**%**
Sex			
	Female	290	66.97
	Male	143	33.03
	Total	433	100.0
Location*			
	City of Port-of-Spain	38	8.78
	City of San Fernando	40	9.24
	Borough of Arima	28	6.47
	Borough of Chaguanas	72	16.62
	Diego Martin	51	11.78
	San Juan/Lavantille	29	6.70
	Tunapuna/Piarco	84	19.40
	Couva/Tabaquite/Talparo	24	5.54
	Penal/Debe	35	8.08
	Other**	32	7.39
	Total	433	100.0
Employed			
	No	176	40.65
	Yes	240	55.43
	Non-response	17	3.92
	Total	433	100.0
Work days/week			
	1–2 days	53	12.24
	3–5 days	178	41.11
	>5 days	51	11.78
	Non-response	151	34.87
	Total	433	100.0
Monthly income***			
	<500 TTD	56	12.93
	500–1,000 TTD	94	21.71
	1,001–1,500 TTD	38	8.78
	1,501–2,000 TTD	53	12.24
	>2,000 TTD	58	13.39
	Non-response	134	30.95
	Total	433	100.0
Pays rent			
	No	14	3.23
	Yes	414	95.61
	Non-response	5	1.16
	Total	433	100.0
Household size			
	1–2 persons	87	20.09
	3–5 persons	248	57.27
	>5 persons	93	21.48
	Non-response	5	1.16
	Total	433	100.0
Presence of chronic illness			
	No	373	86.14
	Yes	60	13.86
	Total	433	100.0

*Area of residence was classified based on municipalities.

**Includes: Borough of Point Fortin, Mayaro/Rio Claro, Sangre Grande, Princess Town, Penal/Debe, Siparia, Tobago.

***Monthly income did not take into consideration donations in the form of clothing, shelter or food assistance.

### Raw food security scores of respondents on each question of the FIES

Table 2 shows the raw scores from respondents on each question of the FIES. A majority indicated having eaten only a few kinds of foods because of a lack of money (mild food insecurity domain) (95.2%) and having eaten less than they thought they should because of a lack of money (moderate domain) (91.2%), while 46% went without eating for a whole day because of a lack of money (severe domain).

**Table 2:** Raw score of responses by all respondents to FIES questions (*n* = 433) by food insecurity domain.

**During the last 12 months, was there a time when……**			***n***	**%**
Item 1	You were worried you would run out of food because of a lack of money?	**Worried**	346	79.91
Item 2	You were unable to eat healthy and nutritious food because of a lack of money?	**Nutritious**	352	81.29
Item 3	You ate only a few kinds of foods because of a lack of money?	**Few kinds**	412	95.15
Item 4	You had to skip a meal because there was not enough money to get food?	**Skip meal**	354	81.76
Item 5	You ate less than you thought you should because of a lack of money?	**Ate less**	395	91.22
Item 6	Your household ran out of food because of a lack of money?	**Runout**	334	77.14
Item 7	You were hungry but did not eat because there was not enough money for food?	**Hungry**	335	77.37
Item 8	You went without eating for a whole day because of a lack of money?	**Whole day**	199	45.96

### Prevalence of food insecurity

**Figure 3:**
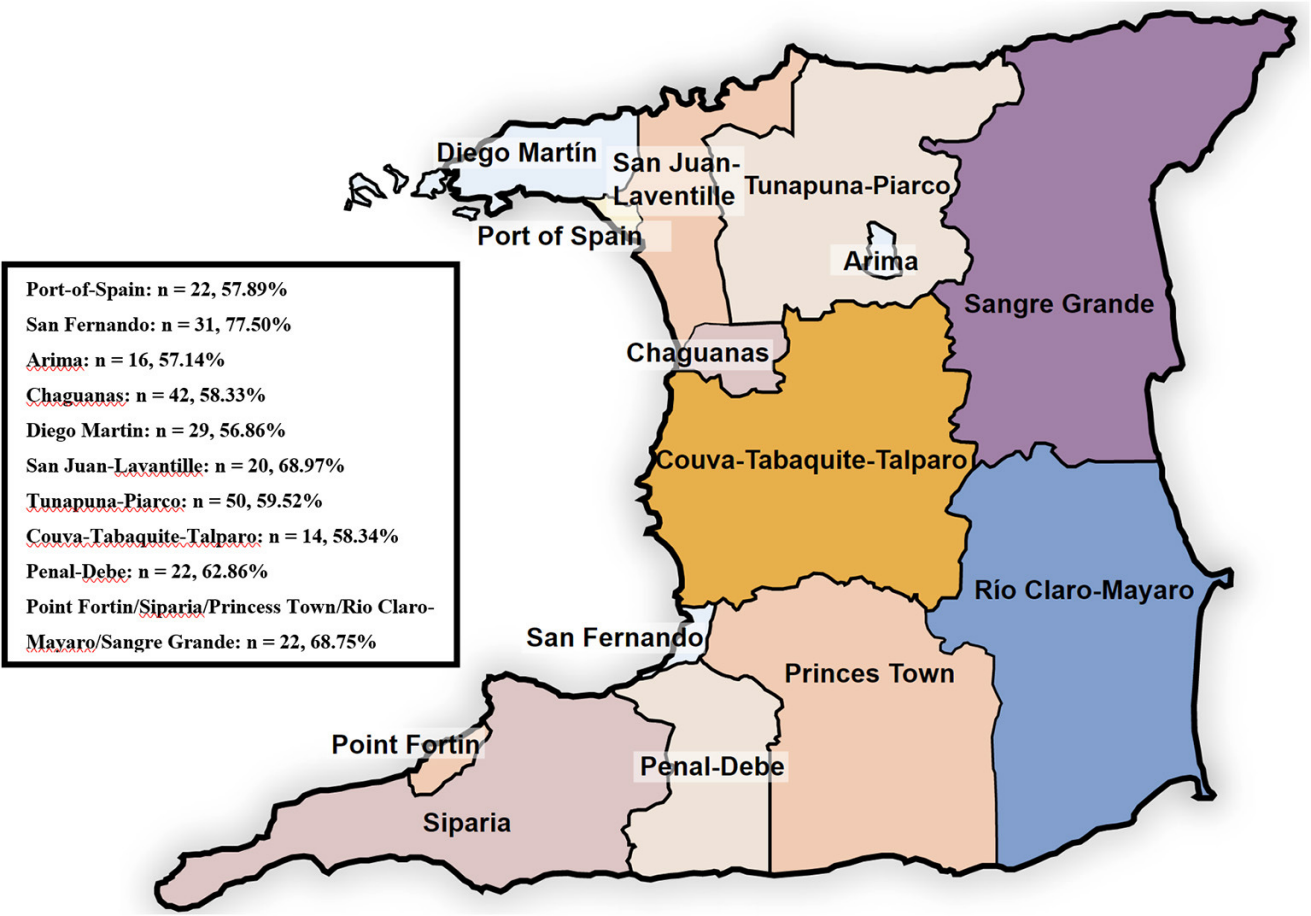
Prevalence of severe food insecurity by location in Trinidad and Tobago.

Table 3 shows the proportions of respondents' food insecurity categories according to sociodemographic and economic factors. Figure 3 presents the prevalence of severe food insecurity in Venezuelan migrants according to their location in Trinidad. Overall, 61.9% of the individuals surveyed were severely food insecure. Significant differences in food security status were not observed when categories of gender (*p* = 0.410), area of residence (*p* = 0.589), number of working days (*p* = 0.409), monthly salary (*p* = 0.338), number of persons living in the household (*p* = 0.536) and the presence of chronic illnesses (*p* = 0.158) were considered. However, significant differences in food security status were observed when categories of employment status (*p* = 0.032) and payment of rent (*p* = 0.005) were considered. There were greater proportions of employed individuals who were food secure to mildly food insecure (17.5%) compared to those who were unemployed (9.1%); and unemployed individuals who were severely food insecure (67.6%) compared to those who were employed (56.7%). There were greater proportions of individuals not paying rent who were food secure to mildly food insecure (42.9%) compared to those who were paying rent (12.6%); and individuals paying rent who were severely food insecure (62.6%) compared to those who were not paying rent (50.0%).

**Table 3:** Proportion of food insecurity experience scale according to sociodemographic and economic factors.

		**Food insecurity experience scale**						**Total**	***p*-value**
		**Food secure to mild**		**Moderate food**		**Severe food**			
		**food insecurity**		**insecurity**		**insecurity**			
	***n***	**%**	***n***	**%**	***n***	**%**	***n***	
Overall		58	13.39	107	24.71	268	61.90	433	
Gender									
	Female	35	12.07	70	24.14	185	63.79	290	0.410
	Male	23	16.08	37	25.88	83	58.04	143	
	Total	58	13.39	107	24.71	268	61.90	433	
Location									
	Port-of-Spain	6	15.79	10	26.32	22	57.89	38	0.589
	San Fernando	2	5.00	7	17.50	31	77.50	40	
	Arima	7	25.00	5	17.86	16	57.14	28	
	Chaguanas	9	12.50	21	29.17	42	58.33	72	
	Diego Martin	8	15.69	14	27.45	29	56.86	51	
	San Juan/Lavantille	5	17.24	4	13.79	20	68.97	29	
	Tunapuna/Piarco	13	15.48	21	25.00	50	59.52	84	
	Couva/Tabaquite/ Talparo	2	8.33	8	33.33	14	58.34	24	
	Penal/Debe	2	5.71	11	31.43	22	62.86	35	
	Other	4	12.50	6	18.75	22	68.75	32	
	Total	58	13.39	107	24.71	268	61.90	433	
Employed									
	No	16	9.09	41	23.30	119	67.61	176	0.032
	Yes	42	17.50	62	25.83	136	56.67	240	
	Non-response	-	-	4	23.53	13	76.47	17	
	Total	58	13.39	107	24.71	268	61.90	433	
Work days/week									
	1–2 days	6	11.32	15	28.30	32	60.38	53	0.409
	3–5 days	24	13.48	49	27.53	105	58.99	178	
	>5 days	11	21.57	9	17.65	31	60.78	51	
	Non-response	17	11.26	34	22.51	100	66.23	151	
	Total	58	13.39	107	24.71	268	61.90	433	
Monthly income									
	<$500 TTD	4	7.14	12	21.43	40	71.43	56	0.338
	$500–$1,000 TTD	13	13.83	25	26.60	56	59.57	94	
	$1,001–$1,500 TTD	6	15.79	9	23.68	23	60.53	38	
	$1,501–$2,000 TTD	4	7.55	15	28.30	34	64.15	53	
	>$2,000 TTD	13	22.41	17	29.31	28	48.28	58	
	Non-response	18	13.43	29	21.64	87	64.93	134	
	Total	58	13.39	107	24.71	268	61.90	433	
Pays rent									
	No	6	42.86	1	7.14	7	50.00	14	0.005
	Yes	52	12.56	103	24.88	259	62.56	414	
	Non-response	-	-	3	60.00	2	0	5	
	Total	58	13.39	107	24.71	268	61.90	433	
Household size									
	1–2 persons	11	12.64	15	17.24	61	70.12	87	0.536
	3–5 persons	33	13.30	67	27.02	148	59.68	248	
	>5 persons	14	15.05	23	24.73	56	60.22	93	
	Non-response	-	-	2	40.00	3	60.00	5	
	Total	58	13.39	107	24.71	268	61.90	433	
Presence of chronic illness									
	No	54	14.48	94	25.20	225	60.32	373	0.158
	Yes	4	6.67	13	21.66	43	71.67	60	
	Total	58	13.39	107	24.71	268	61.90	433	

### Associations between characteristics of respondents and food insecurity

Table 4 summarizes the univariate associations of sociodemographic and economic characteristics with food insecurity in terms of unadjusted odds ratios with 95% confidence intervals; while Table 5 summarizes the multivariate associations of sociodemographic and economic characteristics with food insecurity in terms of adjusted odds ratios with 95% confidence intervals.

**Table 4:** Univariate associations of sociodemographic characteristics with food insecurity in terms of unadjusted odds ratios (OR) and 95% confidence intervals (CI).

**Variables**		**Food insecurity**	
		**(Ref: Food secure to mild food insecurity)**	
		**Unadjusted OR**	**95% CI**
Gender			
	Female****	Ref	Ref
	Male	0.716	0.405–1.265
Location			
	Port-of-Spain****	Ref	Ref
	San Fernando	3.562	0.672–18.886
	Arima	0.562	0.166–1.908
	Chaguanas	1.313	0.429–4.011
	Diego Martin	1.008	0.318–3.193
	San Juan/Lavantille	0.900	0.245–3.301
	Tunapuna/Piarco	1.024	0.357–2.937
	Couva/Tabaquite/Talparo	2.063	0.381–11.176
	Penal/Debe	3.094	0.581–16.476
	Other	1.313	0.336–5.129
Employed			
	No****	Ref	Ref
	Yes	0.471	0.256–0.870
	Non-response	Inf	Inf
Work days/week			
	1–2 days****	Ref	Ref
	3–5 days	0.819	0.316–2.123
	>5 days	0.464	0.158–1.367
	Non-response	1.006	0.375–2.703
Monthly income			
	<500 TTD****	Ref	Ref
	500–1,000 TTD	0.479	0.148–1.550
	1,001–1,500 TTD	0.410	0.107–1.566
	1,501–2,000 TTD	0.942	0.233–3.976
	>2,000 TTD	0.266	0.081–0.875
	Non-response	0.496	0.160–1.537
Pays rent			
	No****	Ref	Ref
	Yes	5.221	1.742–15.649
	Non-response	Inf	Inf
Household size			
	1–2 persons****	Ref	Ref
	3–5 persons	0.943	0.454–1.958
	>5 persons	0.817	0.349–1.911
	Non-response	Inf	Inf
Presence of chronic illness			
	No****	Ref	Ref
	Yes	2.370	0.826–6.803

****Reference category.

Inf: all of the persons in this category were food insecure.

**Table 5:** Multivariate associations of sociodemographic characteristics with food insecurity in terms of adjusted odds ratios (OR) and 95% confidence intervals (CI).

**Variables**		**Food insecurity**	
		**(Ref: Food secure to mild food insecurity)**	
		**Unadjusted OR**	**95% CI**
Gender			
	Female****	Ref	Ref
	Male	0.770	0.395–1.501
Location			
	Port-of-Spain****	Ref	Ref
	San Fernando	3.168	0.538–18.657
	Arima	0.469	0.120–1.835
	Chaguanas	0.956	0.275–3.320
	Diego Martin	0.762	0.210–2.760
	San Juan/Lavantille	0.609	0.141–2.620
	Tunapuna/Piarco	0.876	0.269–2.854
	Couva/Tabaquite/Talparo	1.089	0.174–6.834
	Penal/Debe	3.074	0.503–18.789
	Other	1.317	0.273–6.356
Employed			
	No****	Ref	Ref
	Yes	0.112	0.016–0.763
	Non-response	Inf	Inf
Work days/week			
	1–2 days****	Ref	Ref
	3–5 days	1.732	0.540–5.552
	>5 days	1.237	0.327–4.676
	Non-response	0.442	0.049–3.952
Monthly income			
	<500 TTD****	Ref	Ref
	500–1,000 TTD	0.612	0.162–2.309
	1,001–1,500 TTD	0.597	0.129–2.773
	1,501–2,000 TTD	1.229	0.245–6.172
	>2,000 TTD	0.318	0.075–1.338
	Non-response	0.371	0.083–1.651
Pays rent			
	No****	Ref	Ref
	Yes	7.325	1.965–27.312
	Non-response	Inf	Inf
Household size			
	1–2 persons****	Ref	Ref
	3–5 persons	0.822	0.363–1.861
	>5 persons	0.590	0.225–1.546
	Non-response	Inf	Inf
Presence of chronic illness			
	No****	Ref	Ref
	Yes	2.111	0.688–6.477

****Reference category.

Inf: all of the persons in this category were food insecure.

Univariate analysis based on unadjusted odds ratios with 95% confidence intervals revealed that categories of gender, area of residence, number of working days, monthly income, household size and presence of chronic illnesses were not significantly associated with the food security status of migrants. Notably though, migrants who earned a monthly income over 2,000 TTD were less likely to be food insecure (OR 0.266, 95% CI 0.081–0.875) relative to those who earned under 500 TTD.

Employment status and paying rent were significantly associated with their food security status. Food insecurity was less likely among migrants who were employed (OR 0.471, 95% CI 0.256–0.870) relative to those who were not employed; while food insecurity was more likely among migrants who were paying rent (OR 5.221, 95% CI 1.742–15.649) relative to those not paying rent.

Multivariate analysis based on adjusted odds ratios with 95% confidence intervals showed that categories of gender, area of residence, number of working days, monthly income, household size and presence of chronic illnesses were not significantly associated with the food security status of migrants. However, employment status and paying rent remained significantly associated with their food security status. Food insecurity remained less likely among migrants who were employed (OR 0.112, 95% CI 0.016–0.763) relative to those who were not employed, while food insecurity remained more likely among migrants who were paying rent (OR 7.325, 95% CI 1.965–27.312) relative to those not paying rent.

### Rasch model

#### Fit statistics and reliability of the FIES

The Rasch model analysis sought to detect differences in food security experiences across gender. Table 6 shows the proportion of affirmative responses to FIES items, item severity parameters and item fit statistics grouped by male and female. The INFIT statistics fell within the broadly acceptable range of 0.7–1.3 and none of the outfit statistics were high (>2). This indicates that the FIES measured the same latent trait in both genders. Evaluation of conditional independence showed significant correlations (>0.4) for certain item pairs, namely “worried” and “healthy” for females, “worried” and “healthy” and “worried” and “whole day” for males (results not shown).

**Table 6:** Proportion of affirmative responses to FIES items, item severity parameters and item fit statistics, grouped by sex.

**Item**	**Affirmative responses (%)**	**Severity ±SE**	**Infit**	**Outfit**
**Female**				
Worried	83.8	−0.227 ± 0.225	0.954	1.613
Healthy	84.1	−0.271 ± 0.226	0.889	1.065
Few food	95.9	−2.569 ± 0.407	1.061	0.551
Skipped	81.4	0.064 ± 0.219	0.943	0.624
Ate less	91.7	−1.414 ± 0.278	0.932	1.453
Runout	78.3	0.415 ± 0.213	1.179	0.981
Hungry	77.6	0.491 ± 0.213	0.984	0.801
Whole day	44.8	3.511 ± 0.286	1.018	0.60
**Male**				
Worried	72.0	0.784 ± 0.287	1.036	1.042
Healthy	75.5	0.429 ± 0.296	0.872	0.655
Few food	93.7	−2.777 ± 0.626	1.137	0.537
Skipped	82.5	−0.383 ± 0.326	1.221	1.248
Ate less	90.2	−1.652 ± 0.431	0.930	1.987
Runout	74.8	0.502 ± 0.294	0.999	0.803
Hungry	76.9	0.278 ± 0.301	0.707	0.505
Whole day	48.3	2.818 ± 0.320	1.134	1.246

#### Ordering of FIES items

The item severity parameters proved that only items 6, 7, and 8 performed as expected (see Table 6). Accordingly, item eight (which assesses the most severe experience of respondents going without food for the whole day) was least likely to obtain a “yes” response and thus, measured severe food insecurity consistently. Although the predicted order of difficulty of items 1–5 differed from the actual order on the scale; this indicated disordering of items. However, Table 6 shows that the proportions of affirmative responses to items 1–5 were still higher than those of items measuring severe food insecurity.

#### Prevalence of severe food insecurity across gender

Table 7 shows the results of the Wald test conducted to determine if there were gender-based differences in severity of experiences of food insecurity. The test proved that both genders assigned similar severities to most items in the FIES scale. The exception was item one (worried about running out of food because of lack of money or other resources), for which a statistically significant difference between the genders (*p* = 0.006) was observed. However, this does not introduce bias into the comparisons of food insecurity proportions between genders.

**Table 7:** Wald test applied on item parameters for sex disaggregation.

**Items**	**Wald test *p*-values**
Worried	0.006
Healthy	0.061
s Few food	0.781
Skipped	0.255
Ate less	0.643
Runout	0.810
Hungry	0.564
Whole day	0.107

## Discussion

### Prevalence of food insecurity

This paper examined the food insecurity status of Venezuelan migrants in Trinidad and Tobago. In this study, 61.9% of Venezuelan migrants self-reported experiences that are characterized as severe food insecurity. This substantiates previous findings that convey a high food insecurity prevalence among migrants (7, 8, 47, 48). The behaviors associated with severe food insecurity aligns with previous reports of migrants' survival strategies that reduce access to required food quantity and quality by using as little money as possible (10). The widespread food insecurity among Venezuelan migrants may be attributed to the challenges outlined previously (7–9) and exacerbated by their lack of legal status, which limits meaningful livelihood opportunities in Trinidad and Tobago and predisposes them to exploitation within their existing employment arrangements.

There was a significant association between employment and food security status of Venezuelan migrants, which aligns with previous research that identified unemployment as a contributor to food insecurity in migrants (49, 50). In the present study, a larger proportion of employed migrants were categorized as food secure to mildly food insecure compared to unemployed migrants. Even though the proportion of employed migrants who experienced severe food insecurity was significantly lower than that of unemployed migrants (a difference of ~11%), a large proportion of employed migrants were found to be severely food insecure nonetheless. Only 17.5% of employed migrants fell in the category of food secure to mild food insecurity while more than half (56.7%) experienced severe food insecurity. This indicates that severe food insecurity is still common among employed migrants, though at a lower level than in unemployed migrants.

This may be due to differences in the quality and type of employment that migrants are engaged in. Previous research has evidenced that informal and insecure employment result in Venezuelan migrant families in Colombia having insufficient funds for purchasing food (51). In addition, migrants engaged in casual daily and informal labor experience higher levels of inadequate food intake than those in more secure and regular employment (52). Given that the principal type of employment undertaken by Venezuelan migrants in Trinidad and Tobago is often informal, unskilled, seasonal and low-wage in nature, it is plausible that employed migrants in our study were engaged in labor of this type. Thus, it may be that the wages paid to and disposable incomes of these employed migrants are an insufficient living wage leaving them unable to pay expenses and purchase food, resulting in a high proportion of them being severely food insecure. This attests to the importance of and link between the type of employment (which was not assessed in this study) and migrants' food security status, as informal and casual employment may account for the large proportion of employed migrants experiencing severe food insecurity in our study.

The prevalence of severe food insecurity among employed migrants may also be attributed to the additional financial responsibilities of such individuals. Migrants who earn an income may have to deal with other expenses related to the household such as payment of rent and even transportation, which may further limit the amount of funds available to spend on food. In other words, household dynamics could potentially influence the results, though the dynamics were not assessed in this study. The understanding of migrant household dynamics is an under-researched area that should be examined to better understand the factors that give rise to the association between food insecurity, employment and migrants living in large households.

Despite this apparent contradiction, the present study's findings indicate that food insecurity is less likely among migrants who are employed when compared to those unemployed. Considering that unemployment contributes to food insecurity (49, 50), it is expected that the opposite would be true. Employment can increase the income available for spending on food, thereby making food security more likely among employed migrants

Food security status of Venezuelan migrants was also associated with payment of rent, with a larger proportion of migrants paying rent being severely food insecure than those who were not paying rent. It was also found that migrants who paid rent were more likely to be food insecure than those who were not paying rent. This parallels extant research which showed that migrants living in rented accommodation were more likely to experience food insecurity than those who were owners of their residence (50). This may be because the additional expense of paying rent limits the amount of money available for purchasing food, as was the case with Venezuelan migrants in Colombia (51). Paying rent is often an expense that cannot be delayed as failing to do so or tardiness in payment increases the risk of eviction and homelessness (52). As such, paying rent may be prioritized in migrant communities in order to retain a place of shelter, while purchasing food may be sacrificed. Univariate and multivariate analyses yielded that the other socio-demographic and economic characteristics assessed were not significantly associated with migrants' food security status.

### Rasch model and FIES suitability

The academic community recognizes food insecurity as a process in which severity increases from worry, to changes in diet quality, to reductions in quantity and ultimately to severe hunger (53). The participants' ordering of responses to items 1–5 of the FIES differs from this common understanding of food insecurity. According to prior users of the FIES, respondents may assign item 2's (“nutritious”) severity to the moderate domain instead of the mild domain of food insecurity (54). Additionally, qualitative research has revealed that local cultural understanding of “food” in question two of the FIES can give rise to misunderstanding as “food that gives energy” related to farming activities (54, 55). Linguistic differences in the local translation of FIES questions from English to Spanish and difference in food cultures may also have contributed to these discrepancies (55). Nevertheless, the proportions of affirmative responses to items 1–5 were higher than for items measuring severe food insecurity. Moreover, questions 6–8, which measure severe food insecurity, performed as expected. This aligns with other studies, which propose FIES items 6–8 for use as global anchors (40). This strengthens the position that the FIES is largely consistent and well-suited for easy mobilizing to conduct spot checks among vulnerable populations such as migrants in measuring severe food insecurity.

### Policy implications

These findings provide a rapid assessment that can be used to galvanize international, national and community-level stakeholders to devise responses to assist migrants experiencing challenges related to food security. Second, measures like the FIES can help develop or oversee in-country programs, especially in developing countries where resources are scarce and efficient use of these resources is essential. Lastly, rapid assessments from measures like FIES and other experience-based food security measures can help to identify locations and characteristics of migrants that are at greatest risk to ensure that these vulnerable groups are identified by the PADF and implementing partner NGOs for better targeting of planned interventions.

While this study is one of the few that assesses food insecurity in Venezuelan migrants in Trinidad and Tobago and provides evidence on the suitability of the FIES as a tool, a number of limitations should be noted. This study was conducted during the early stages of the COVID-19 pandemic in Trinidad and Tobago, which was under a nation-wide lockdown. Consequently, a virtual platform was necessary for collecting data as mobility was severely inhibited. The online and social media recruitment methods employed in this study limited involvement of migrants residing in rural areas with marginal access to service providers, NGOs and referral agencies and those without a mobile phone and internet connectivity. Additionally, because of the highly mobile nature of migrants, the “12-month reference period” specified in the FIES may have included time spent in a food insecure status in Venezuela as the survey did not consider the date of the migrants' arrivals in Trinidad and Tobago. Further, this study also failed to address the association of migrants' dietary and food purchasing habits and household dynamics with food insecurity.

## Conclusion

This study revealed a high prevalence of severe food insecurity among Venezuelan migrants and asylum seekers in Trinidad and Tobago and demonstrated usefulness of the recently developed experience-based scale, FIES, as part of a rapid online assessment of individual food security situation of migrants in Trinidad and Tobago. This study provides one of the few working examples of the use of FIES as an experience-based food security measure in the Caribbean region that allows for a rapid assessment of a marginalized group in a timely manner.

## Data availability statement

The raw data supporting the conclusions of this article will be made available by the authors, without undue reservation.

## Ethics statement

The studies involving human participants were reviewed and approved by the Campus Research Ethics Committee, The University of the West Indies, St. Augustine Campus. Written informed consent for participation was not required for this study in accordance with the national legislation and the institutional requirements.

## Author contributions

AV and IF-G: conceived and designed the study, collected, interpreted and discussed the data, wrote, and discussed the manuscript. BB: contributed importantly to the analysis and the interpretation of data, discussed, and reviewed the manuscript. MM assisted with finalizing the references, preparation of the manuscript, and reviewing the final manuscript. All authors contributed to the article and approved the submitted version.

## Funding

This study was fully supported by the Pan American Development Foundation and funded by the United States Government. The funding body was not involved in the design of the study, collection, analysis, and interpretation of data, nor the writing the manuscript. The content is solely the responsibility of the authors and does not necessarily represent the views of the Pan American Development Foundation.

## Conflict of interest

The authors declare that the research was conducted in the absence of any commercial or financial relationships that could be construed as a potential conflict of interest.

## Publisher's note

All claims expressed in this article are solely those of the authors and do not necessarily represent those of their affiliated organizations, or those of the publisher, the editors and the reviewers. Any product that may be evaluated in this article, or claim that may be made by its manufacturer, is not guaranteed or endorsed by the publisher.

## Abbreviations

FIES: Food Insecurity Experience Scale
MRAS, Migrants: Refugees and Asylum Seekers
TTD: Trinidad and Tobago Dollars.
